# A deficiency of uPAR alters endothelial angiogenic function and cell morphology

**DOI:** 10.1186/2045-824X-3-10

**Published:** 2011-05-02

**Authors:** Rashna D Balsara, Reid Merryman, Farhaad Virjee, Claire Northway, Francis J Castellino, Victoria A Ploplis

**Affiliations:** 1 W. M. Keck Center for Transgene Research, University of Notre Dame, 230 Raclin-Carmichael Hall, Notre Dame, Indiana 46556, USA; 2 Department of Chemistry and Biochemistry, University of Notre Dame, 230 Raclin-Carmichael Hall, Notre Dame, Indiana 46556, USA

## Abstract

The angiogenic potential of a cell requires dynamic reorganization of the cytoskeletal architecture that involves the interaction of urokinase-type plasminogen activator receptor (uPAR) with the extracellular matrix. This study focuses on the effect of uPAR deficiency (uPAR^-/-^) on angiogenic function and associated cytoskeletal organization. Utilizing murine endothelial cells, it was observed that adhesion, migration, proliferation, and capillary tube formation were altered in uPAR^-/- ^cells compared to wild-type (WT) cells. On a vitronectin (Vn) matrix, uPAR^-/- ^cells acquired a "fried egg" morphology characterized by circular actin organization and lack of lamellipodia formation. The up-regulation of β1 integrin, FAK(P-Tyr925), and paxillin (P-Tyr118), and decreased Rac1 activation, suggested increased focal adhesions, but delayed focal adhesion turnover in uPAR^-/- ^cells. This accounted for the enhanced adhesion, but attenuated migration, on Vn. VEGF-enriched Matrigel implants from uPAR^-/- ^mice demonstrated a lack of mature vessel formation compared to WT mice. Collectively, these results indicate that a uPAR deficiency leads to decreased angiogenic functions of endothelial cells.

## Introduction

Neovascularization, by way of angiogenesis, involves a series of tightly regulated cellular processes. As a pathological event that is required for growth and survival of tumor cells, angiogenic signals consist of growth factors released in the microenvironment by the hypoxic tumor. These growth factors activate quiescent endothelial cells (ECs), leading to disruption of cell-extracellular matrix (ECM) contacts. Subsequently, the ECs undergo concerted changes in morphology and cytoskeletal configuration [1]. These processes enable growth factor-induced migration [2], followed by adhesion [3], proliferation, and formation of a new vascular lumen, eventually leading to development of a blood vessel [4]. The initial disruption of the EC-ECM contact requires degradation of the ECM, which is facilitated by a variety of proteases. The urokinase-plasminogen activator receptor (uPAR) binds to urokinase-plasminogen activator (uPA) [5,6], which in-turn localizes the activation of plasminogen (Pg) to the extracellular protease, plasmin (Pm) [7]. Pm then catalyzes degradation of the ECM and also activates other proteases, which together facilitate EC migration. Additionally, uPAR, by lateral interactions with its transmembrane partners, e.g., integrins [8] and low-density lipoprotein receptor-related protein (LRP), functionally orchestrates bidirectional signaling events that affect migration, adhesion, and proliferation [9]. The ability of uPAR to interact with cytoskeletal components, such as vinculin, Rac, and focal adhesion kinase (FAK), at sites of EC-ECM contacts, strongly implicates its role in cytoskeletal rearrangement [10-12].

uPAR can directly interact with vitronectin (Vn), and this interaction may be enhanced by uPA, thus promoting cellular events leading to angiogenesis [8]. Several studies have shown that increased expression of uPAR, which is upregulated in different cancers [13-18], results in increased adhesion to Vn. Hence, down-regulating uPAR expression would potentially not only disrupt cell-associated uPA, but also binding to matrix proteins, thereby suppressing tumor growth and invasion. A uPAR deficiency would also affect reciprocal molecular binding of integrins to ECM proteins, modulating signaling events and cytoskeleton morphology. Thus, loss of uPAR function disrupts the integrated processes of pericellular proteolysis, cell adhesion and migration, and downstream signaling events. This is confirmed in studies that showed that attenuated uPAR expression in tumor cell lines inhibited tumor cell migration and invasiveness, and led to inactivation of ERK1/2 signaling and rearrangement of the cytoskeleton architecture [18,19]. Further, silencing uPAR expression in CFPAC-1 and PANC-1 pancreatic ductal adenocarcinoma cell lines significantly inhibited cell proliferation and migration with an increase in apoptosis [19]. On the other hand overexpression of uPAR in HEK293 cells increased adhesion to Vn, with marked display of protrusions and lamellipodia, compared to mock-transfected cells [20,21]. Thus, it appears that direct interaction of uPAR with Vn leads to matrix adhesion, followed by lateral engagement with integrins, which activates downstream events such as changes in cell morphology, migration, and signal transduction [20].

It is apparent that changes in the physiological levels of uPAR have biological consequences in this regard. Increased expression of uPAR enhanced adhesive and migratory properties of cells accompanied by increased ERK1/2 activation [20], whereas diminished uPAR levels in cancer cells proved to be detrimental for tumor growth and invasiveness [22]. However, implications of diminished uPAR expression, and its effect on the angiogenic functions of cells, are not well documented. Since uPAR plays an important role in angiogenesis, as well as coordinating various cellular responses, such as interaction with matrices, signaling, and cell morphology, a total deficiency of uPAR on these processes was comprehensively evaluated utilizing physiologically relevant primary ECs isolated from aortas of wild-type (WT) and uPAR^-/- ^mice.

## Materials and methods

### Materials

Complete cell culture medium for ECs consisted of RPMI 1640 (Mediatech, Herndon, VA), 20% fetal bovine serum (Invitrogen, Carlsbad, CA), 1% antibiotic/antimycotic mixture (1000 units of penicillin, 0.1 mg of streptomycin, 0.25 μg amphotericin B) (Sigma, Saint Louis, MO), 50 μg/ml endothelial growth factor supplement (BD Biosciences, San Jose, CA), 2 mM glutamine (Mediatech), 0.1 mM amino acids (Invitrogen), and 1 μl/ml β-mercaptoethanol (Invitrogen). Primary antibodies utilized were rabbit anti-human-total FAK, rabbit anti-human-total paxillin, rabbit anti-human-phospho-FAK, rabbit anti-human RhoA, rabbit anti-human Rac1/2/3 (L129), and rabbit anti-human STAT1 were from Cell Signaling Technology (Danvers, MA). Armenian hamster anti-mouse integrin β1 monoclonal antibody, goat anti-human integrin β3, and mouse anti-porcine tubulin monoclonal antibody were from Santa Cruz Biotechnology (Santa Cruz, CA). Rabbit anti-human paxillin(P-Tyr118) polyclonal antibody conjugated to Alexa Fluor 488 was from Invitrogen. For actin visualization, phalloidin conjugated to either Alexa Fluor 488 or 594 (Invitrogen) was utilized. Where required the secondary antibodies were HRP-conjugated goat anti-rabbit IgG (Cell Signaling Technology), goat anti-mouse IgM, and goat anti-armenian hamster (Santa Cruz Biotechnology). Mouse anti-human uPA and mouse anti-human vinculin antibodies were from Abcam (Cambridge, MA) and rabbit anti-rat PAI-1 from American Diagnostica (Stamford, CT). Vitronectin was from Sigma and fibronectin and rat-tail type I collagen was from BD Biosciences. Rabbit anti-human microtubulin polyclonal antibody was kindly provided by Dr. Holly Goodson (University of Notre Dame, IN, USA).

### Mice

Mice with a homozygous deficiency of urokinase-type plasminogen activator receptor (uPAR^-/-^) have previously been described [23]. The uPAR^-/- ^mice utilized in this study lacked the second and fifth exon of the uPAR gene resulting in complete inactivation of the gene product. Wild type mice (C57BL/6J) were obtained from the Jackson Laboratory (Bar Harbor, ME) and were used as controls. Male mice between 8 and 12 wks of age were utilized for this study. Mice were anesthetized intraperitoneally with a rodent mixture (0.015 mg of xylazine, 0.075 mg of ketamine, and 0.0025 mg of aceprozamine/g body weight). Experimental protocols were approved by the Institutional Animal Care and Use Committee of the University of Notre Dame.

## Methods

### Isolation of EC

Aortic ECs were isolated as previously described [24]. The aortas were cut into 10 pieces and then opened longitudinally. Each segment was placed lumen side down on a collagen gel and incubated for 36 hr. Fresh media was added and cells were allowed to become confluent (7-10 days). Automated EC purification was based on selecting a subset of cells that are positive for CD105 and/or CD106 expression utilizing a RoboSep (Stem Cell Research, Vancouver, Canada). All cell culture experiments were performed at 37°C in a humidified 6.5% CO_2 _incubator.

### Adhesion assay

Twenty four-well plates were coated with Vn (1.5 μg/ml) or Fn (10 μg/ml) at 4°C overnight. Plates coated with collagen (1 mg/ml) were incubated for 1 hr at RT. The wells were aspirated and the plates were treated with 1% BSA for 1 hr at 37°C to prevent non-specific binding. Wells coated with 40 μg/ml of BSA served as controls for the adhesion assay. ECs were placed in serum-free medium for 1 hr to induce quiescence. EC density was adjusted to 5 × 10^4 ^cells/ml in RPMI/0.2% BSA, and 1 ml was added to each well. After the designated incubation time, the cells were placed on a Jitterbug™ model 130000 (Boekel Scientific, Feasterville, PA) for 2 minutes and then washed with PBS to remove non-adherent cells. The adherent cells were fixed with cytofix, stained with Hematoxylin (Vector Laboratories), viewed with a Nikon Eclipse TE200 microscope, and imaged using the SPOT camera and the SPOT advanced version 4.0.9 software (Diagnostic Instruments, Inc.). Cell counts were determined in triplicate using a 40× objective (three fields/well).

### Migration assay

Migration assays were performed on 6-well Vn- (1.5 ug/ml) or collagen- (1 mg/ml) coated plates. 6 × 10^5 ^quiescent cells in RPMI were added to each well and allowed to adhere overnight. The next day, a scratch was induced with a 200 μl tip, the cells washed with PBS and then incubated in RPMI containing 10 ng/ml of VEGF as a chemoattractant. Images were captured immediately after scratch induction and 24 hr after scratch induction with a Nikon Eclipse TE200 microscope utilizing a 10× objective, the SPOT camera, and SPOT advanced version 4.0.9 software. The number of cells that had migrated to the scratched area was counted. For uPA/PAI-1 colocalization staining, WT and uPAR^-/- ^ECs were seeded on Vn-coated 2-well multi-chambered slides (Grace Biolabs, Bend, OR) and allowed to reach confluency. A scratch was then induced, and migration allowed to occur for 24 hr. The cells were fixed with 4% paraformaldehyde and stained for PAI-1 and uPA (as described for immunofluorescence).

### Proliferation assay

Cells were resuspended at 1 × 10^5 ^cells/ml in complete medium. An aliquot of 1 ml was added to Vn-coated or collagen-coated 6-well plates. The cells were then incubated for 24 hr. The medium was then removed, fresh complete medium without EC growth factor supplement was added, and the plates incubated at 37°C. Total cell counts were performed at 24 and 48 hr. For this, the medium was removed, and the wells were rinsed with PBS. Adherent cells were detached either with collagenase (Invitrogen) or trypsin-EDTA, stained with trypan blue (Sigma), and counted using a hemocytometer.

### Western blot

WT and uPAR^-/- ^ECs that were allowed to adhere on Vn for 4 hr were detached by trypsin-EDTA for 5 min and then lysed in cell lysis buffer (Cell Signaling Technology) for 10 min on ice. The cell lysates were centrifuged and total protein concentration of the supernatant was determined by the BCA assay (Pierce, Rockford, IL). Supernatants were fractionated on 10% SDS-PAGE gels, blotted on polyvinylidene difluoride membranes (Osmonics Inc.), and immunoassayed according to the manufacturer's protocol. Densitometric analyses of Western autoradiograms were performed using the Scion program downloaded from NIH (available on the World Wide Web at http://www.scioncorp.com).

### Immunoprecipitation

For detection of integrins β1, β3, and FAK(P-Tyr925), immunoprecipitation of 100 μg of WT and uPAR^-/- ^cell lysates from ECs adherent on Vn for 4 hr was performed using an antigen specific antibody and incubated overnight at 4°C. Lysates were then gently rocked for 3 hr with protein A beads (Pierce) at 4°C. The mixture was centrifuged and washed 5× in lysis buffer, suspended in SDS-PAGE loading buffer, and resolved in a 10% polyacrylamide gel. Western blots were then performed.

### Immunofluorescence microscopy

WT and uPAR^-/- ^cells plated on Vn or collagen-coated multi-chambered wells (Grace Biolabs) were fixed with either 4% paraformaldehyde for 10 min. or ice-cold methanol for 5 min. Cells not fixed in methanol were permeabilized with 0.1% Triton X-100, and blocked with 10% normal serum (Jackson Immunoresearch, West Grove, PA) followed by treatment with Image-iT FX signal enhancer (Invitrogen). Cells were incubated with primary antibody, washed, and then incubated with the appropriate Alexa Fluor secondary antibody (488 or 594) (Molecular Probes). Cells stained for actin or paxillin(P-Tyr118) were permeabilized with 0.1% Triton X-100/PBS at RT for 5 min, washed 3×, and incubated with Image-iT FX for 30 min. The conjugated primary antibody diluted (1:500 for phalloidin, 1:50 for phospho-pax) in 1% BSA/PBS was then added and incubated overnight at 4°; C. After labeling, the slides were rinsed and cover slipped. Coverslips were mounted with ProLong Gold antifade reagent containing DAPI (Invitrogen). Images were captured using a Nikon Eclipse TE2000-U with the BD CARV spinning disk confocal unit and images were acquired using Metamorph 7.0 software.

### Rac activity assay

Rac1 activity was measured utilizing the Rac1 activation assay kit according to the manufacturer's protocol (Thermo Scientific, Rockford, IL). WT and uPAR^-/- ^ECs adherent on Vn for 4 hr were harvested in lysis/binding/wash buffer containing 1× protease inhibitor cocktail (Thermo Scientific). Briefly, one Immobilized Glutathione SwellGell Disc was placed in a spin tube for each genotype and to that was added 20 μg of GST-human Pak1-PBD. Immediately 800 μg of cell lysates was added, the spin tube vortexed, and then incubated at 4°C with gentle rocking for 1 hr. The resin was washed 3× with the lysis/binding/wash buffer and the Rac activation pull-down reaction was retrieved by adding 50 μl of 2× SDS Sample buffer containing 1 part β-mercaptoethanol and centrifuging at 7,200 × g for 2 min. The samples were fractionated by SDS-PAGE using a 12% gel and subjected to immunoblot analysis utilizing anti-Rac 1 mouse monoclonal antibody. The secondary antibody was goat anti-mouse HRP conjugated IgG and detection was by chemiluminescent. The presence of active Rac was determined by the appearance of the 22 kDa Rac-GTP band.

### Matrigel assay

An in vivo angiogenesis assay utilizing Matrigel Basement Membrane Matrix (BD Biosciences) was performed. Matrigel solution was supplemented with VEGF (10 ng/ml) and 0.4 ml was administered to WT and uPAR^-/- ^mice (3 mice/genotype) by dorsal subcutaneous injection. All mice utilized in this experiment were 8-10 wk old males. The implants were removed at day 14 and fixed in PLP (Paraformaldehyde/Lysine/Periodate). The excised plugs were stained for smooth muscle α-actin by first permeabilizing with 0.05% Triton X-100, followed by blocking with normal goat serum for 30 min with agitation. The gels were incubated with a mouse monoclonal antibody against human smooth muscle α-actin (Sigma) at 4°; C/overnight, washed, stained with Alexa Fluor 488 (Invitrogen) and then visualized utilizing an Olympus FV 1000 Laser Scanning Confocal microscope with ASW software (NDIIF, University of Notre Dame, USA).

### In vitro sprout formation assay

The ability of WT and uPAR^-/- ^ECs to undergo tubulogenesis, in vitro, was determined as previously described [25]. Microcarrier cytodex (Sigma) beads were mixed with 5 × 10^6 ^cells and seeded in a fibrinogen (ERL, South Bend, IN) gel in a 2-well multi-chambered slide. Polymerization of fibrin was initiated by adding 0.48 U/ml thrombin (ERL) for 5 min at RT. To each well 1 ml of complete RPMI was added and incubated for 1 h at 37°; C, after which the medium was aspirated and 1 ml of complete RPMI in the presence or absence of 10 ng/ml VEGF was added. The mixture was allowed to incubate for either 24 or 96 hr at 37°; C/6.5% CO_2_, after which the gel was fixed with 1% paraformaldehdye/PBS for 3 h at 4°; C. The cells were permeabilized with 0.3% Triton X-100 for 5 min at room temperature and then treated with Image-iT Fx Signal Enhancer (Invitrogen) for 30 min. The cells were washed with PBS 3× and blocked with a serum-free protein block (DAKO, Denmark) for 5 min. The cells were then incubated overnight at 4°; C with phalloidin Alexa Fluor 594 (Invitrogen) to stain for actin. The next day the cells were washed 3× with PBS and the gels were mounted with DAPI containing ProLong Antifade reagent (Invitrogen).

### Quantification of vessels in vitro

High resolution images of the beads (up to 10 beads/well of each genotype/treatment) were acquired using a Nikon TE 2000 S fluorescence microscope. The objective used was 100× (Nikon). The images were taken as a z series stack, which allowed the inclusion of all sprouts. The software interface NIS Nikon Elements was used to capture the images as well as to quantify the number of cells adherent per bead (performed by counting the nuclei on a bead), sprouts per bead, and the sprout length. After a 96 h incubation, images were acquired using a 20× ELWD objective. All experiments were repeated three times.

### Statistical analysis

All experiments were performed at least three times. The data are represented as the mean ± SEM and *p *values of ≤ 0.05 obtained using Student's *t *test were considered to be statistically significant.

## Results

### A total uPAR deficiency perturbs adhesion, migration, and proliferation of ECs

**Figure 1:**
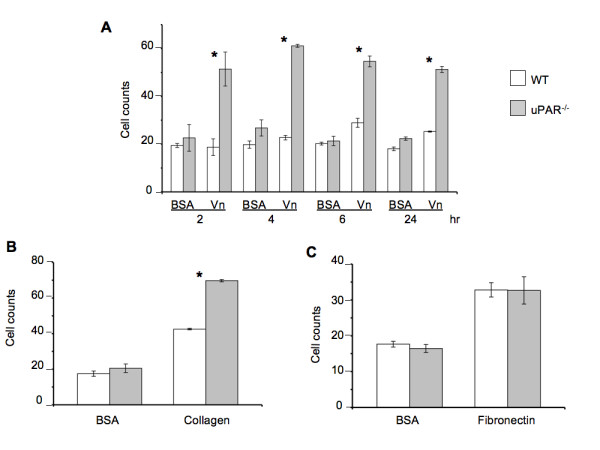
**An uPAR deficiency alters cell adhesion to Vn and collagen**: (**A**) uPAR deficiency promotes EC adhesion to Vn as observed by enhanced adhesion of uPAR^-/- ^ECs compared to WT cells. WT and uPAR^-/- ^cells on BSA serve as a control for this assay. (**B**) Enhanced adhesion of uPAR^-/- ^ECs on collagen at 4 hr. (**C**) Adhesion of uPAR^-/- ^ECs is similar to WT cells when plated on fibronectin. BSA served as a negative control matrix. The cell counts represent the mean ± SEM of three independent assays each performed in triplicate (40× field). Significance levels (*) indicates *p *value of < 0.05 between WT and uPAR^-/- ^ECs.

The ability of ECs to adhere to the ECM and reorganize their cell morphology is a critical step in angiogenesis [26]. uPAR has been implicated in pathological angiogenesis [9] and, due to the ability of the receptor to functionally interact with ECM [27], integrins [28,29], and G-protein-coupled receptors [30], uPAR can facilitate different angiogenic events. WT and uPAR^-/- ^ECs were utilized to study the processes associated with angiogenesis, i.e., adhesion, migration, and proliferation. It was observed that uPAR^-/- ^ECs adhered more strongly on Vn and collagen compared to WT cells (Figure 1A,B). uPAR^-/- ^ECs displayed markedly increased adhesion on Vn, which peaked at 4 hr, but was statistically significant even after 24 hr (Figure 1A). However, adhesion of uPAR^-/- ^ECs to fibronectin was unaffected (Figure 1C).

**Figure 2:**
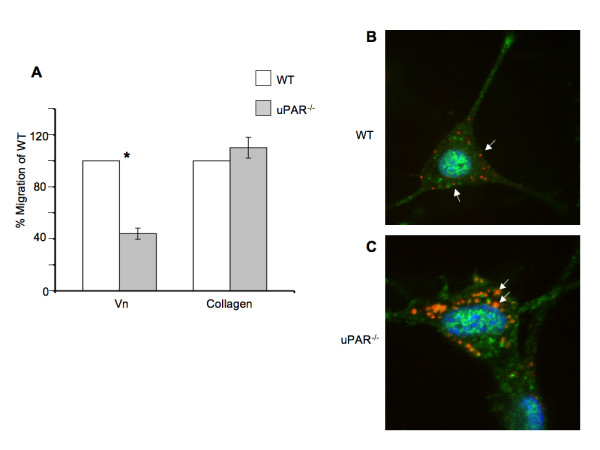
**Endothelial cell migration on Vn and uPA/PAI-1 co-localization are affected by a uPAR^-/- ^deficiency**: (**A**) Absence of uPAR expression decreased EC migration on Vn but not on collagen. Quiescent WT and uPAR^-/- ^ECs were plated to confluency on Vn- and collagen-coated 6-well dishes and a scratch induced. Migration was induced by the presence of VEGF (10 ng/ml) and images of the scratch area were acquired immediately after the scratch and after 24 hr of incubation at 37°; C/6.5% CO_2_. The number of cells that had migrated in the scratch area was counted and graphed as percent of migrated WT cells. The graph represents the mean ± SEM of three independent assays each performed in triplicate. Significance levels (*) indicates *p *value of < 0.05 between WT and uPAR^-/- ^ECs. (**B**) uPA/PAI-1 co-localization is cytoplasmic in migratory uPAR^-/- ^ECs. Fixed cells were stained with antibodies against uPA (green, Alexa Fluor 488) and PAI-1 (red, Alexa Fluor 647). Co-localization of uPA/PAI-1 in WT cells was observed along the focal adhesions or cell membrane (arrows). (**C**) In the uPAR^-/- ^ECs uPA/PAI-1 co-localization was observed as extensive blobs within the cytoplasm (arrows).

Since cell migration and proliferation are coupled to adhesive properties of cells on the ECM, migration and proliferation assays on Vn and collagen were performed. A scratch assay was employed to assess the cellular motility of ECs from both genotypes in the presence of Vascular Endothelial Cell Growth Factor (VEGF), which is a major facilitator of both physiological and pathological angiogenesis. Cell migration of uPAR^-/- ^ECs was diminished 60% compared to WT cells when plated on Vn (Figure 2A). However, migratory function of the uPAR^-/- ^ECs was not affected on collagen. ECM proteolysis is controlled by the inhibitor, plasminogen activator inhibitor-1 (PAI-1), which forms a 1:1 complex with uPA bound to uPAR, and catalyzes the vesicular internalization of the uPA/PAI-1 complex. This results in eventual degradation of both uPA and PAI-1 [31]. Primary ECs express both uPA and PAI-1 [24], and since migration of uPAR^-/- ^cells is impaired on Vn, co-localization of uPA and PAI-1 by immunofluorescence on cells migrating in the presence of VEGF was performed. In migrating WT cells, uPA and PAI-1 co-localization was observed along the focal adhesions at the advancing front of the cell (indicated by arrows) (Figure 2B). Co-localization of uPA/PAI-1 in the uPAR^-/- ^ECs was observed abundantly within the cytoplasm (indicated by arrows), with very little staining at focal adhesion points (Figure 2C). ERK1/2 activation levels were not affected in uPAR^-/- ^ECs compared to WT cells (data not shown) indicating that changes in angiogenic function in these cells are regulated by other signaling pathways.

**Figure 3:**
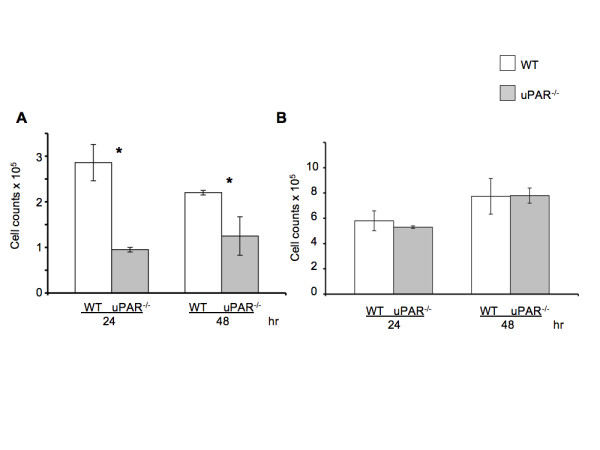
**uPAR^-/- ^ECs exhibited altered proliferation on Vn**: (**A**) WT and uPAR^-/- ^ECs were seeded on Vn-coated plates at a density of 1 × 10^5 ^cells/ml as described. Graph represents total cell counts measured at 24 and 48 hr (mean ± SEM) of three independent assays. At both time points proliferation of uPAR^-/- ^ECs was significantly diminished compared to WT cells. *p *values between WT and uPAR^-/- ^ECs at both time points were <0.05. (**B**) Proliferation of WT and uPAR^-/- ^ECs is similar on collagen-coated plates at 24 and 48 hr.

The proliferative ability of uPAR^-/- ^ECs plated on Vn (Figure 3A) was decreased compared to WT cells, but not when plated on collagen (Figure 3B) at 24 and 48 hr. On Vn-coated plates, WT cell counts increased by ~3-fold from its initial plating density of 1 × 10^5 ^cells at 24 hr compared to uPAR^-/- ^ECs. At 48 hr the uPAR^-/- ^cell counts have marginally increased compared to WT cells on Vn-coated plates. The slight decrease in WT cell counts at 48 hr when Vn is utilized as the matrix could be attributed to a functional plasminogen activation system. Since the proliferation is a long-term assay, it is possible that proteolysis of Vn could lead to cell detachment. Therefore, while adhesion of uPAR^-/- ^cells on Vn and collagen was increased, the migratory and proliferative properties of the cells were only affected when the cells were plated on Vn, and not on collagen.

### Altered morphology of uPAR^-/- ^ECs is Vn-dependent

**Figure 4:**
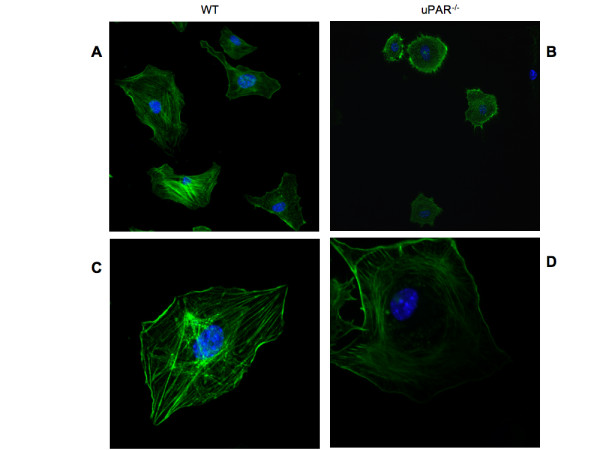
**Actin cytoskeleton organization is circular in uPAR^-/- ^ECs**: Lack of uPAR expression induces changes in cell morphology and actin cytoskeleton. Quiescent WT (**A, C**) and uPAR^-/- ^(**B, D**) ECs were plated on Vn and allowed to adhere for 4 hr and then stained for actin with phalloidin-FITC. Cells stained for actin (green) and the nucleus (blue) were imaged using a confocal microscope. Images obtained using a 30× objective (**A, B**) revealed morphological differences between WT and uPAR^-/- ^ECs. Images at higher magnification (100× objective) (**C, D**) demonstrated that while the WT cells showed polarized actin formation, the uPAR^-/- ^ECs maintained an atypical concentric actin organization.

Our data demonstrated that uPAR^-/- ^cells had stronger adhesive properties with concomitant decreased migratory and proliferative abilities when plated on Vn compared to WT cells. Since cell morphology on the ECM is a critical parameter for determining cell growth and apoptosis, the actin organization of uPAR^-/- ^and WT ECs was analyzed when cultured on both Vn and collagen. Immunofluorescence studies revealed that the uPAR^-/- ^ECs showed typical "fried egg" morphology with a lack of actin polarization and lamellipodia formation when plated on Vn compared to WT cells (Figure 4A,B). WT cells showed the expected elongated cell shape with polarized actin and several lamellipodia extensions (Figure 4A,C). The actin architecture in uPAR^-/- ^ECs was observed as a circular network of filaments around the nucleus with radial filaments ending towards the edges of the plasma membrane (Figure 4B,D). uPAR^-/- ^cells grown on collagen did not exhibit the "fried egg" morphology and were morphologically similar to WT cells (data not shown).

Microtubules functionally interact with the actin cytoskeleton and together they organize the cytoskeleton architecture, thus facilitating cell migration [32-34]. To determine whether lack of uPAR has an influence on microtubule configuration, WT and uPAR^-/- ^ECs adherent to Vn and collagen were stained with an antibody specific for polymerized α-tubulin (kindly provided by Dr. Holly Goodson). When WT and uPAR^-/- ^ECs were plated on Vn, no differences in the morphology of microtubule organization were noted. Both genotypes exhibited a prominent microtubule-organizing center (MTOC) with the microtubules growing radially towards the cell periphery (Additional File 1, Figure S1A,B). However, on collagen the microtubulin organizations in uPAR^-/- ^ECs appeared to be arranged in parallel bundles, as opposed to the radial petal-like configuration observed in WT cells (Additional File 1, Figure S1C,D). This may be a function of microtubules in uPAR^-/- ^cells having a greater number of contact points with the cell cortex, as the petal motif in the WT cells arise from the convergence of microtubules to a few specific points on the membrane.

### Effect of uPAR-deficiency on cytoskeletal-associated signaling in ECs

**Figure 5:**
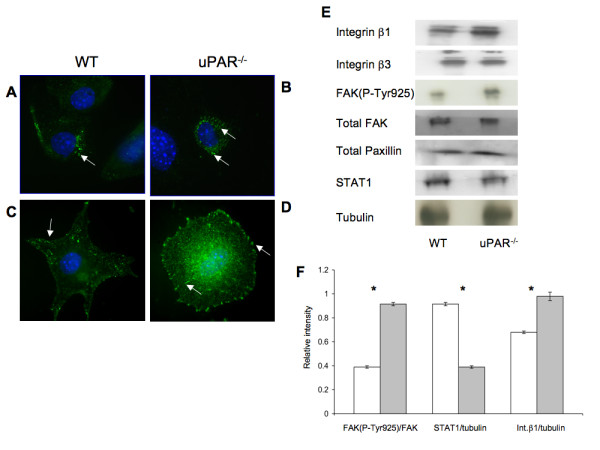
**uPAR^-/- ^ECs demonstrated changes in focal adhesion proteins FAK(P-Tyr925), Pax(P-Tyr118), integrins, and signal transduction**: (**A**) WT cells adherent on Vn for 4 hr were stained for FAK(P-Tyr925) (green) and the nucleus (blue) and were imaged using an 100× objective. FAK(P-Tyr925) was found to be localized on focal adhesions (arrow) in WT cells. (**B**) uPAR^-/- ^ECs stained for FAK(P-Tyr925) demonstrated that FAK(P-Tyr925) was present not only along the cellular membrane (arrow) but also centrally in the cytoplasm (arrow). (**C**) Similar to (**A**), WT cells adherent on Vn were stained for Pax(P-Tyr118) (green) and DNA (blue). Images (100× objective) demonstrated that Pax(P-Tyr118) is present mainly along the focal adhesions and lamellipodia (arrow) in WT cells. Robust lamellipodia formation was observed. (**D**) Pax(P-Tyr118) (green) localization in uPAR^-/- ^ECs adherent on Vn occurred along the focal adhesions (arrow) of the circularly shaped cell as well as centrally within the cytoplasm (arrow). Lack of lamelllipodia formation was observed in uPAR^-/- ^ECs (**B, D**). (**E**) Immunoblot analyses of focal adhesion proteins and STAT1. Quiescent WT and uPAR^-/- ^ECs were seeded on Vn-coated 6-well plates and allowed to adhere for 4 hr. The medium was aspirated and the cell lysates were utilized for analyses. Levels of integrins β1, β3, and FAK(P-Tyr925) were determined by IP of 100 μg of cell lysates. Levels of integrin β1 and FAK(P-Tyr925) were enhanced in uPAR^-/- ^ECs compared to WT cells, while levels of integrin β3, total FAK, and paxillin were similar between WT and uPAR^-/- ^ECs. uPAR^-/- ^ECs showed decreased levels of STAT1 compared to WT cells. Tubulin served as a loading control. (**F**) The graph shows the levels of FAK(P-Tyr925/FAK), STAT1/tubulin, and Integrin β1/tubulin between WT and uPAR^-/- ^ECs. The values obtained represent the mean ± SEM of three independent assays. Significance levels (*) indicates *p *value of < 0.05 between WT and uPAR^-/- ^cells.

Since adhesion of ECs to the ECM requires formation of focal adhesions that form cell-matrix junctions that aid in anchoring cytoskeletal proteins to the matrix [35], focal adhesion kinase (FAK) and paxillin, which are key components of focal adhesions that become phosphorylated when cells adhere to the ECM [36], were examined. The cellular distributions of FAK(P-Tyr925) and Pax(P-Tyr118) were evaluated in WT and uPAR^-/- ^ECs. After 4 hr adhesion on Vn, FAK(P-Tyr925) was observed as small punctate signals, mainly in the cytoplasmic region of the WT cells, with very little staining observed on the membrane edge (Figure 5A). However, in uPAR^-/- ^ECs FAK(P-Tyr925) was prominently localized in the central adhesion regions with some localization on the membrane edge (Figure 5B). Additionally, elevated levels of FAK(P-Tyr925) were observed in uPAR^-/- ^ECs by immunoblotting (Figure 5E) and densitometric analyses (Figure 5F). Phosphorylation of FAK at Tyr925 as well as at other sites (Tyr407, 576, 577, and 861) is Src-dependent [37-40] and is essential for regulating F-actin assembly and maintaining cell adhesion dynamics [41]. Phosphorylation of endogenous FAK at Tyr925 is known to exclude FAK from focal adhesion points [42], which is observed in WT cells. Endogenous FAK(P-Tyr925) has been implicated in focal adhesion disassembly resulting in normal focal adhesion turnover and hence a migratory phenotype for the cell. On the other hand hyperphosphorylation of FAK(P-Tyr925) in uPAR^-/- ^EC resulted in increased cytoplasmic localization of FAK, but not total exclusion of FAK from focal adhesions. This suggests that the levels of FAK(P-Tyr925) in focal adhesions are still sufficient to promote adhesion of the cells to Vn.

Paxillin is another major component of the focal adhesion complex playing a pivotal role in cell attachment and motility. This adaptor protein is phosphorylated at different sites. Phosphorylation at Tyr118 is catalyzed by FAK [43] and this step is important for coordinating formation of focal adhesions and associated actin network [44]. Immunofluorescence staining was performed on WT and uPAR^-/- ^ECs after 4 hr adhesion on Vn to determine the localization and distribution of Pax(P-Tyr118). In WT cells, Pax(P-Tyr118) was present as plaques in the lamellipodia protrusions, whereas in the uPAR^-/- ^ECs there is complete lack of lammellipodia and the Pax(P-Tyr118) was observed as focal adhesions around the cell periphery and central region (Figure 5C,D). Localization of Pax(P-Tyr118) at focal adhesions, and actin organization, are inter-dependent processes [44,45], and tethered to the actin filaments [46]. Therefore, changes to the actin organization can affect the localization of paxillin. Since in uPAR^-/- ^ECs actin organization is circular, corresponding peripheral and central circular localizations of Pax(P-Tyr118) were observed in these cells (Figure 5D). In adherent WT cells that showed polarized actin organization and robust lamellipodia formation, Pax(P-Tyr118)-containing focal adhesions were associated with lamellipodia protrusions.

It has been reported that modification of cytoskeletal proteins affects paxillin expression [45]. However, changes in the actin network in uPAR^-/- ^cells had little effect on the expression of paxillin (Figure 5E). Since vinculin is another component of focal adhesions and is associated with paxillin [47], adherent cells were immunostained for vinculin after 4 hr. It was observed that in both WT and uPAR^-/- ^ECs plated on Vn, cellular localization of vinculin was more perinuclear (Additional File 2, Figure S2A,B). Cellular localization of vinculin on WT and uPAR^-/- ^ECs plated on collagen was associated with focal adhesions (Additional File 2, Figure S2C,D). Thus, it appears that subcellular localization of vinculin is independent of actin organization or uPAR, but dependent on the type of ECM on which the cells are plated.

The Rho family of GTPases, viz., Rac1, cdc42, and RhoA, play an important role in actin cytoskeleton regulation of lamellipodia and filopodia formation, thus controlling cell adhesion, motility, and growth [48,49]. Rac activation is dependent on the interaction of uPAR with Vn followed by protrusive activity and lamellipodia formation [10]. To investigate whether Rac activation is affected in uPAR^-/- ^cells adherent on Vn, conditions where cell morphology was affected, levels of activated Rac were determined on ECs after 4 hr of adhesion. As observed in Additional File 3, figure S3A, GTP-loaded Rac was diminished in uPAR^-/- ^ECs, but was present in WT cells. Since functioning of Rac and RhoA is interconnected, and RhoA regulates formation of actin bundles and focal adhesions, the spatial arrangement of Rho was examined in WT and uPAR^-/- ^cells on Vn and collagen. In WT cells, RhoA is located centrally throughout the cell when plated on VN and collagen (Additional File 3, Figure S3B,D). On the other hand, in the uPAR^-/- ^ECs, RhoA appears to be present on the membrane when adhered to collagen, but is perinuclear when plated on Vn (Additional File 3, Figure S3E,C). The central localization of Rho in WT cells is consistent with its function of generating tension and stabilizing adhesions [50].

ECs interact with Vn via members of the integrin family of ECM receptors, which in-turn are known to mediate tyrosine phosphorylation of FAK, thus regulating cell adhesion and migration [51]. In particular, it is known that ECs predominantly bind to Vn via the α_5_β_1 _and α_V_β_3 _integrins [52], and that uPAR is known to associate with β1 and β3 integrins [53]. Given our observation that uPAR^-/- ^ECs demonstrate perturbations in adhesive properties, key components of the cytoskeletal components, and focal adhesions, expression levels of β1 and β3 integrins in cells adherent to Vn for 4 hr were evaluated. It was observed that β1 integrin levels were elevated in uPAR^-/- ^ECs (Figure 5E,F), whereas expression of β3 integrins was similar to WT cells (Figure 5E). These results suggest that absence of uPAR increased expression of β1 integrin leading to higher levels of Vn cell surface receptors and elevated adhesion of uPAR^-/- ^cells to Vn. It has been shown that STAT1 plays an important role in cell adhesion in different cell types and that STAT1^-/- ^embryonic fibroblasts demonstrated higher levels of adhesiveness and decreased migration on fibronectin compared to WT cells [54,55]. Therefore, STAT1 levels were evaluated in WT and uPAR^-/- ^ECs after 4 hr of adhesion on Vn. STAT1 levels in uPAR^-/- ^ECs were lower compared to WT cells (Figure 5E,F).

### Sprout formation and capillary morphogenesis is impaired in uPAR^-/- ^ECs

**Figure 6:**
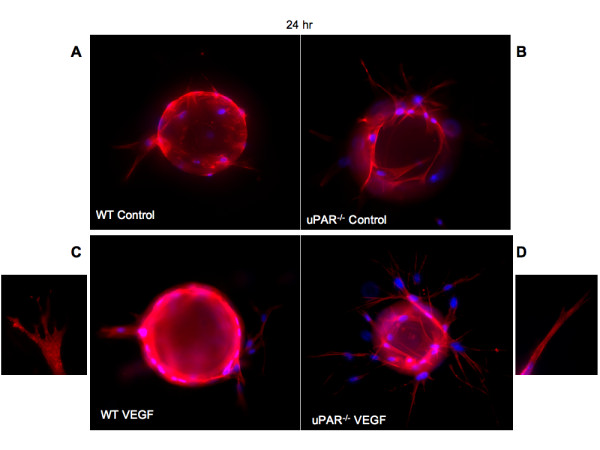
**In vitro angiogenesis is impaired in uPAR^-/- ^ECs**: (**A**) WT cells on cytodex beads and embedded in a fibrin matrix were stained for actin using phalloidin-TRITC (red) and DNA (blue) and imaged using a 100× objective. After 24 hr incubation the bead was surrounded by adherent cells which were elongated. (**B**) Enhanced elongation of sprouts was observed from uPAR^-/- ^ECs adherent on the beads and these sprouts appeared to be thinner. (**C**) Sprout elongation by WT cells in the presence of 10 ng/ml of VEGF. Boxed area shows the development of robust lamellipodia formation of WT cells. (**D**) Elongation process after 24 hr for uPAR^-/- ^ECs in the presence of 10 ng/ml of VEGF. Boxed area shows that the sprouts derived from the uPAR^-/- ^ECs are thinner and show truncated lamellipodia formation.

**Figure 7:**
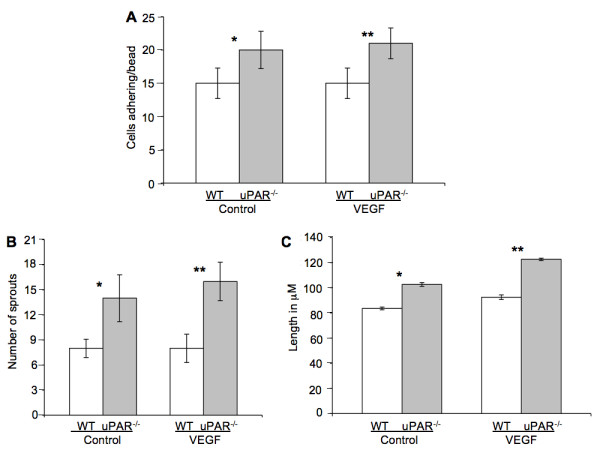
**Quantification of in vitro tube formation assay**: (**A**) Quantification of the number of cells adherent on the beads. Increased number of uPAR^-/- ^ECs adhered to the beads compared to WT cells, both in the absence or presence of VEGF. (**B**) Increased number of sprouts/bead was observed in uPAR^-/- ^cells in the absence or presence of VEGF compared to WT cells. (**C**) uPAR^-/- ^cells demonstrated increased sprout length compared to WT ECs in the absence and presence of VEGF. Ten beads/treatment/experiment/genotype were analyzed and the graph represents the mean ± SEM of three independent assays. (*) Denotes significance between control WT and uPAR^-/- ^ECs and (**) identifies significance between WT and uPAR^-/- ^ECs in the presence of VEGF. *p *value were < 0.05 between WT and uPAR^-/- ^cells.

We show that uPAR^-/- ^cells are altered in their angiogenic properties, e.g., proliferation, migration, and adhesion compared to WT cells when plated on Vn. To determine whether these altered angiogenic functions translate into an inability of the uPAR^-/- ^ECs to form capillary sprouts and lumen, a microcarrier-based fibrin gel angiogenesis assay was performed in vitro. WT and uPAR^-/- ^ECs adherent to collagen-coated cytodex beads were embedded in a fibrin matrix [56,57], which is known to induce EC tube formation in the presence or absence of VEGF. Capillary morphogenesis was observed after 24 and 96 hr incubation and stained for actin and nuclei. After 24 hr, sprouting or elongation of WT and uPAR^-/- ^ECs was observed in the absence (Figure 6A,B) or presence of VEGF (Figure 6C,D). However, the number of uPAR^-/- ^cells adhering to the microcarrier bead was higher (Figure 7A) and in consonance with our observation that these cells adhere more on collagen compared to WT cells (Figure 1B). Assessment of sprout number and length of sprouts per bead revealed that, in the absence of VEGF, uPAR^-/- ^ECs developed more sprouts and the sprout length was longer compared to WT cells (Figure 7B,C). At higher magnification it was observed that WT ECs developed robust filopodia or finger-like projections at the end of the sprout after 24 hr, but the uPAR^-/- ^ECs developed sprouts that were thinner with poorly developed filopodia structures (Figure 6, boxed areas). The presence of VEGF (10 ng/ml) did not appear to significantly enhance the number of cells adherent to the beads or the number and length of sprouts of WT cells (Figure 7A,B,C). However, sprout length of uPAR^-/- ^ECs was increased in the presence of VEGF (Figure 7C).

**Figure 8:**
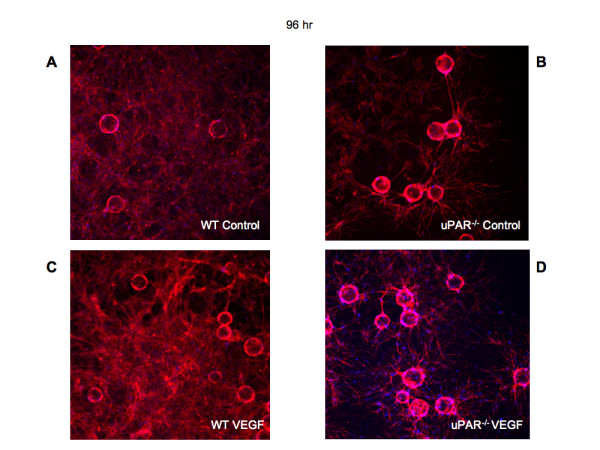
**Tubulogenesis is impaired in uPAR^-/- ^ECs**: Bead-based fibrin gel angiogenesis was observed at 96 hr and the cells were stained for actin using phalloidin-TRITC (red) and DNA (blue) and imaged using a 20× objective. Formation of capillary-like structures at 96 hr in WT cells in control (**A**) assay and in the presence of 10 ng/ml VEGF was observed (**C**). In beads plated with uPAR^-/- ^ECs, highly branched sprouts were observed but were not organized to form capillary-like structures in the absence or presence of VEGF (**B, D**).

After 96 hr it was observed that WT ECs were able to organize into lumen-containing capillary-like structures (Figure 8A,C). However, uPAR^-/- ^cells were unable to orchestrate tubulogenesis, and lumen formation was scarce and irregular in shape (Figure 8B,D). The uPAR^-/- ^ECs appeared to grow longer sprouts that are highly branched and that could contact another vessel, but were unable to undergo normal anastomosis and form lumen-like structures, as was the case with WT cells. Thus, it is concluded that depletion of uPAR in angiogenically functional ECs disrupts cell migration, proliferation, adhesion, and anastomosis, events that are crucial for new blood vessel growth in vivo.

### uPAR^-/- ^mice demonstrate impaired neovascularization in vivo

**Figure 9:**
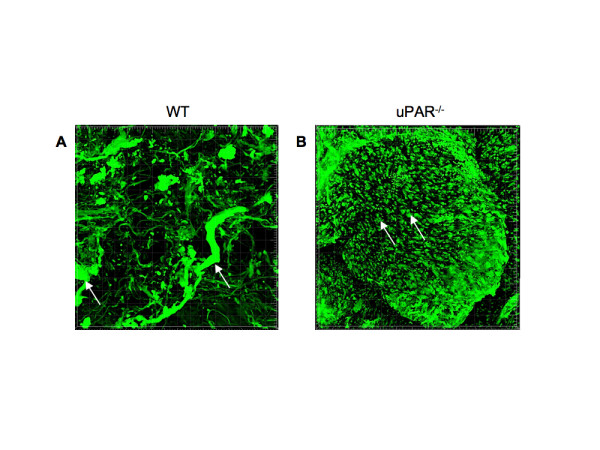
**Effect of a uPAR deficiency on angiogenesis in an in vivo Matrigel assay**: Matrigel containing 10 ng/ml VEGF was implanted s.c. and recovered on day 14 after implantation, fixed, and stained for smooth muscle α-actin to observe capillary formation. (**A**) Representative image of Matrigel implant excised from WT mouse shows the presence of robust and well-developed capillaries (arrows). (**B**) Representative image of Matrigel implant excised from uPAR^-/- ^mouse shows the presence of several truncated structures that have not been organized to form a functional capillary (arrows).

In order to further demonstrate that uPAR is required for promoting normal angiogenesis, an experimental model of in vivo blood vessel formation was performed. Matrigel matrix containing VEGF (10 ng/ml) was injected subcutaneously in WT and uPAR^-/- ^mice and incubated for 14 days. The Matrigel plug was excised and stained for α-smooth muscle actin to detect formation of new blood vessels in the Matrigel implants. Immunofluorescence imaging revealed that implants from WT mice showed the presence of robustly formed capillaries, whereas implants from uPAR^-/- ^mice contained several tiny punctate capillary-like vessels (Figure 9A,B). These results indicate that the absence of uPAR did not support normal vessel formation within the Matrigel plug which was further supported by in vitro observations demonstrating altered proliferation and migratory properties which would contribute to an inability of these cells to organize into functional blood vessels.

## Discussion

**Figure 10:**
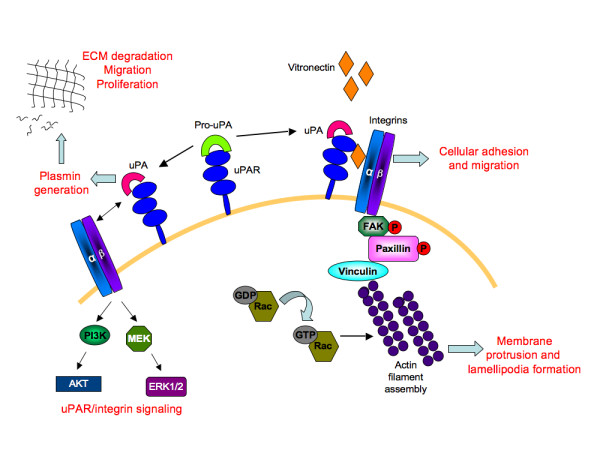
**A simplified schematic depiction of the role of uPAR in angiogenesis**: The uPAR/Pro-uPA interaction leads to the generation of active uPA on the cell surface. This complex binds to vitronectin in the extracellular matrix, allowing interaction with its transmembrane partners, the α/β integrins. This leads to a cascade of activation events resulting in tyrosine phosphorylation of focal adhesion kinase (FAK) and paxillin molecules. Through recruitment of other adaptor molecules, such as Src and p130Cas-CRK complex (not shown), Rac is activated. Activated Rac then induces actin filament assembly leading to membrane protrusion and motility. Formation of focal adhesion complexes enables cellular adhesion and migration. The uPA/uPAR complex also generates the serine protease, plasmin that degrades the extracellular matrix (ECM) thereby stimulating conditions for migration and proliferation. The transmembrane partnership between uPA/uPAR and α/β integrins also activates mitogen activated kinase signaling molecules, MEK and ERK1/2, as well as the phosphoinositide 3-kinase (PI3K)/Akt signaling axis. Thus uPAR-dependent multiple signaling events regulates cellular adhesion, proliferation, and migration, events associated with angiogenesis.

The uPAR protein is a versatile molecule that coordinates a number of cellular processes [9,58]. These include its interaction with Vn [8], which is required for maintaining normal physiological activities, such as haemostasis [59] and angiogenesis [60] (Figure 10). Vn levels are known to increase during pathological conditions of rheumatoid arthritis [61] and tumor invasion [62]. The role of uPAR in pathological angiogenesis is well established and down-regulating uPAR expression in cancerous cells attenuates tumor growth, accompanied by decreased cellular migration, invasion, and adhesion [63-65]. In this study, uPAR^-/- ^ECs demonstrated enhanced adhesion compared to WT cells when tested on Vn and collagen, but not on fibronectin. It is known that uPAR is required for cells to adhere to Vn and collagen [27,28], which then regulates integrin-mediated signaling [66]. In the absence of uPAR the ECs show a "fried egg" morphology and lack lamellipodia formation when plated on Vn compared to WT cells. However these cells exhibited normal cell morphology on collagen. Thus, further evaluations of different focal adhesion and signaling molecules were performed on ECs adherent on Vn. It was also observed that levels of β1-integrins in uPAR^-/- ^ECs were increased after adhesion on Vn with a concomitant increase in focal adhesion protein FAK(P-Tyr925). Thus in the absence of uPAR, which would otherwise bind to Vn, the presence of enhanced levels of β1-integrin and FAK(P-Tyr925) in uPAR^-/- ^ECs is most likely responsible for enhanced adhesion of these cells to Vn. Indeed, it has been reported that HEK293 cells not expressing uPAR are capable of integrin-mediated adhesion to Vn but fail to do so under integrin blocking conditions [21]. Aberrant integrin and FAK signaling is known to facilitate tumor cell invasion and metastasis. However, its activation in each cell type is interpreted differently and current knowledge regarding integrin/FAK signaling is contradictory and dependent on interactions with other focal adhesion proteins, such as paxillin, p130Cas, GRb2, and Shc [67,68]. As shown schematically in Figure 10, interaction of uPA with uPAR results in generation of plasmin that degrades the extracellular matrix facilitating cellular migration and proliferation. Even though the uPAR^-/- ^ECs exhibited increased adhesion on Vn and collagen, a total uPAR deficiency would lead to compromised uPA/uPAR interaction, thus preventing uPA/uPAR-dependent proteolysis that would otherwise positively influence cellular migration and proliferation.

It is proposed that the circular actin organization observed when uPAR^-/- ^ECs were plated on Vn, and the associated increased levels of other proteins, such as FAK(P-Tyr925) and Paxillin, could be responsible for the uPAR^-/- ^EC phenotypes on Vn. Mechanistically, the interaction of uPAR with Vn leads to Rac activation, which in turn affects actin organization and formation of protrusions in an uPA-independent manner in Swiss 3T3 cells [10]. The uPAR^-/- ^ECs adherent on Vn lacked GTP-loaded Rac. RhoA localization in these cells was mostly present at the peripheral edge when cells adhered to collagen and perinuclear when adhered to Vn. This suggests that in order to have normal associated cytoskeletal structures and actin organization when bound to Vn the cells must express uPAR. However, the perinuclear localization of vinculin in WT and uPAR^-/- ^ECs, which is another important component of the focal adhesion complex and is known to complex with FAK and paxillin, was dependent on Vn. Vinculin at focal adhesions was observed only when WT and uPAR^-/- ^ECs were plated on collagen and hence its localization is uPAR-independent.

uPAR^-/- ^ECs expressed decreased levels of STAT1 relative to WT cells. It has been reported that absence of STAT1 signaling is accompanied by increased cell adhesion on fibronectin, but poor migration [69], an observation that was similar to that observed in uPAR^-/- ^ECs. Since cell adhesion and cytoskeleton morphology is intrinsically linked with migration, the decreased migration of uPAR^-/- ^ECs on Vn is probably associated with the perturbed cytoskeletal organization and engagement of focal adhesion proteins of these cells to the matrix. Gondi et al., (2004) [70] have shown that RNAi-mediated down regulation of uPAR expression in SNB19 human glioblastoma cells inhibited tumor cell migration, proliferation, and invasion, but had no effect on phosphorylation of ERK1/2 and FAK. Our observations regarding P-ERK1/2 are consistent with those observed in the uPAR down-regulated SNB19 cells, but in uPAR^-/- ^ECs FAK phosphorylation was increased. This could be due to differences in cell types such as the ECs utilized in this study. Similarly, knockdown of uPAR expression in PC3 prostrate cancer cell lines also diminished cell proliferation and inhibited Matrigel invasion of PC3 cells without affecting ERK1/2 activation [17].

uPAR^-/- ^ECs also exhibited diminished capacity to undergo tubulogenesis both in vitro and in vivo. The inability of the uPAR^-/- ^ECs to form lumen-like structures in a fibrin matrix could not be related to differences in VEGF receptor signaling between WT and uPAR^-/- ^ECs, as the VEGF receptor-1 (Flt-1) levels and its phosphorylated form were similar in the two genotypes (data not shown). Previous studies have demonstrated that these ECs do not express VEGFR-2 [24]. It was demonstrated in systemic sclerosis microvascular ECs (SSc MVECs) that full length uPAR is required for cdc42- and Rac-mediated cytoskeletal organization. SSc MVECs express a truncated form of uPAR that lacks the D1 domain and are incapable of forming tubular-like structures. Instead, elongated cells with very little evidence of capillary-like structures were observed [71]. Similarly, Matrigel implants excised from uPAR^-/- ^mice and stained for α-smooth muscle actin revealed a lack of robustly formed blood vessels. For several years it was thought that the uPAR^-/- ^mice do not have physiological abnormalities as they appeared normal and were fertile. However, several recent investigations have documented that uPAR^-/- ^mice are deficient in several physiological functions. Bone homeostasis in uPAR^-/- ^mice is affected due to increased bone mass, increased osteogenic potential of osteoblasts, decreased osteoclast formation, and altered cytoskeleton organization in matured osteoclasts characterized by actin rings and podosomes clusters when cultured on Vn [72]. Wei et al., (2008) [73] demonstrated that a concerted signaling pathway involving uPAR/α_v_β_3_/Vn is required for development of podocyte foot process effacement and proteinuria, thus implicating the involvement of uPAR in remodeling of the kidney barrier function. Additionally, uPAR is involved in the recruitment and infiltration of macrophages into the lungs and inflamed peritoneal cavity of mice challenged with *Streptococcus pneumoniae *[74,75]. Interestingly, although uPAR^-/- ^mice survive through adulthood they exhibit increased anxiety behavior and are susceptible to spontaneous seizure activity that are thought to be due to decreased levels of GABA-immunoreactive interneurons in the brain cortex thus causing changes in circuit organization and behavior [76]. Thus the uPAR protein is a very versatile molecule and binding to ECM (mainly Vn) can modulate several physiological processes in a proteolytic-independent manner.

## Abbreviations List

CFPAC-1: cystic fibrosis pancreatic adenocarcinoma cell; cdc42: cell division cycle 42; EC: endothelial cell; ECM: extracellular matrix; ERK1/2: extracellular signal-regulated kinase; FAK: focal adhesion kinase; Flt-1: fms-like tyrosine kinase 1; GABA: gamma-aminobutyrylcholine; GPI: glycosylphosphatidylinositol; HEK293: human embryonic kidney cell line 293; LRP: low-density lipoprotein receptor-related protein; MTOC: microtubule-organizing center; PAI-1: plasminogen activator inhibitor-1; PANC-1: human pancreatic carcinoma epithelial-like cell line-1; Pax: paxillin; Pg: plasminogen; Pm: plasmin; Rac and Rho: Ras-related small GTPase; SSc MVEC: systemic sclerosis microvascular endothelial cell; STAT1: signal transducer and activator of transcription 1; uPA: urokinase type plasminogen activator; uPAR: urokinase-type plasminogen activator receptor; VEGF: vascular endothelial growth factor; VEGFR2: vascular endothelial growth factor receptor 2; Vn: vitronectin; WT: wild-type.

## Competing interests

The authors declare that they have no competing interests.

## Authors' contributions

Most of the experiments were performed by RB and RM. In vitro tube formation assays were performed by FV. All authors contributed to analysis and interpretation of data. All authors read and approved the final manuscript.

## Supplementary Material

Additional file 1 **A Deficiency of uPAR Alters Endothelial Angiogenic Function and Cell Morphology**. ***Figure S1. Immunostaining analysis of microtubulin in WT and uPAR^-/- ^ECs: ***Cells adherent on Vn- and collagen-coated oncyte wells for 4 hr were stained with a microtubulin specific antibody (red) and DAPI nuclear stain (blue) and images acquired using a 100× objective. WT (**A**) and uPAR^-/- ^(**B**) ECs plated on Vn show similar microtubule organization. (**C**) WT cells plated on collagen showed similar microtubule organization as observed on Vn. (**D**) However, uPAR^-/- ^cells plated on collagen contain parallel bundles of microtubules that closely approach the plasma membrane, an arrangement not observed in WT cells. Click here for file

Additional file 2 **A Deficiency of uPAR Alters Endothelial Angiogenic Function and Cell Morphology**. ***Figure S2. Perinuclear localization of vinculin in WT and uPAR^-/- ^ECs when plated on Vn*: **WT and uPAR^-/- ^cells adherent on Vn-coated and collagen-coated oncyte wells for 4 hr were stained with vinculin (red), and DAPI nuclear stain (blue) and images acquired using a 100× objective. It was observed that when WT (**A**) and uPAR^-/- ^(**B**) ECs are plated on Vn, vinculin is localized around the nucleus. When WT (**C**) and uPAR^-/- ^(**D**) ECs were plated on collagen, vinculin was localized on focal adhesion points. Click here for file

Additional file 3 **A Deficiency of uPAR Alters Endothelial Angiogenic Function and Cell Morphology**. ***Figure S3. Effect of uPAR deficiency on Rac activity and RhoA localization*: **(**A**) Cell lysates (800 μg) from WT and uPAR^-/- ^ECs adherent on Vn for 4 hr were assayed to determine Rac activity. PAK-1 PBD agarose bound to Rac-GTP was fractionated on a 12% SDS-PAGE gel followed by immunoblotting for Rac. It was observed that uPAR^-/- ^ECs lack endogenous Rac activity compared to WT cells even though total Rac levels were similar. (**B, C, D, E**) WT and uPAR^-/- ^cells adherent on collagen- or Vn-coated oncyte wells for 4 hr were stained with an antibody to RhoA (red), and DAPI nuclear stain (blue) and images acquired using a 100× objective. It was observed that in WT cells on Vn (**B**) RhoA is concentrated in a 'halo' around the nucleus while in uPAR^-/- ^ECs (**C**) RhoA is localized along the membrane. (**D**) WT cells on collagen demonstrated cytosolic punctate staining of RhoA. (**E**) Perinuclear localization of RhoA is observed in uPAR^-/- ^cells adherent on collagen. Click here for file
