# Supplementary figures and images for: A deficiency of uPAR alters endothelial angiogenic function and cell morphology
Source: Vasc Cell. 2011 May 2;3:10. doi: 10.1186/2045-824X-3-10 (PMC3105951; doi:10.1186/2045-824X-3-10)

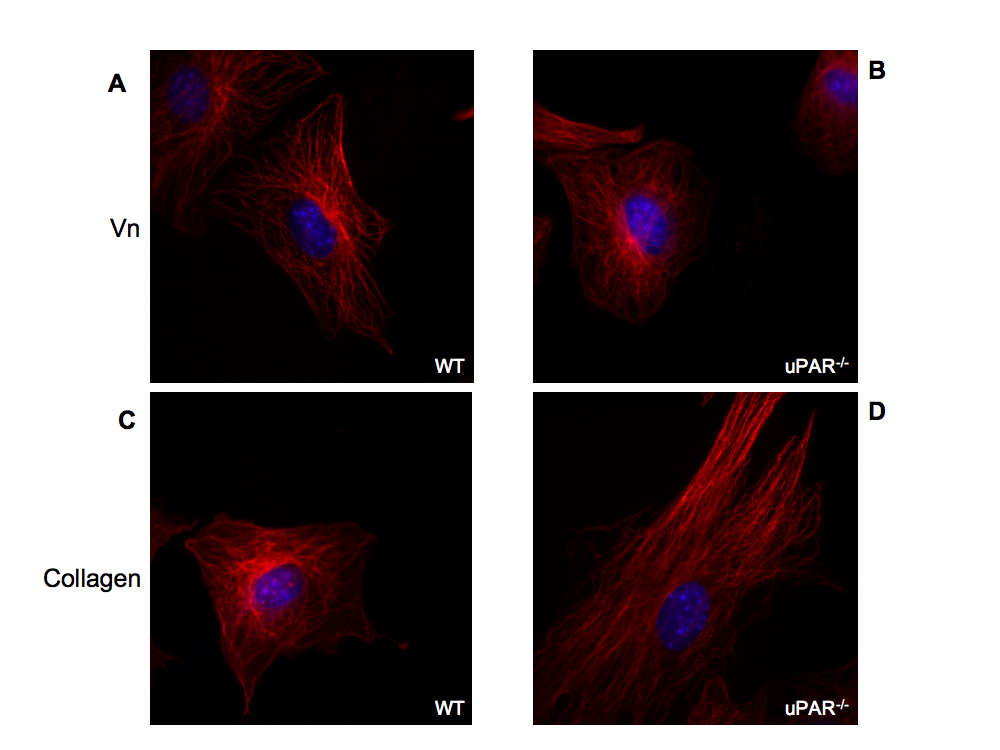

Supplement: Additional file 1 — A Deficiency of uPAR Alters Endothelial Angiogenic Function and Cell Morphology. Figure S1. Immunostaining analysis of microtubulin in WT and uPAR-/- ECs: Cells adherent on Vn- and collagen-coated oncyte wells for 4 hr were stained with a microtubulin specific antibody (red) and DAPI nuclear stain (blue) and images acquired using a 100× objective. WT (A) and uPAR-/- (B) ECs plated on Vn show similar microtubule organization. (C) WT cells plated on collagen showed similar microtubule organization as observed on Vn. (D) However, uPAR-/- cells plated on collagen contain parallel bundles of microtubules that closely approach the plasma membrane, an arrangement not observed in WT cells. [file 2045-824X-3-10-S1.TIFF]

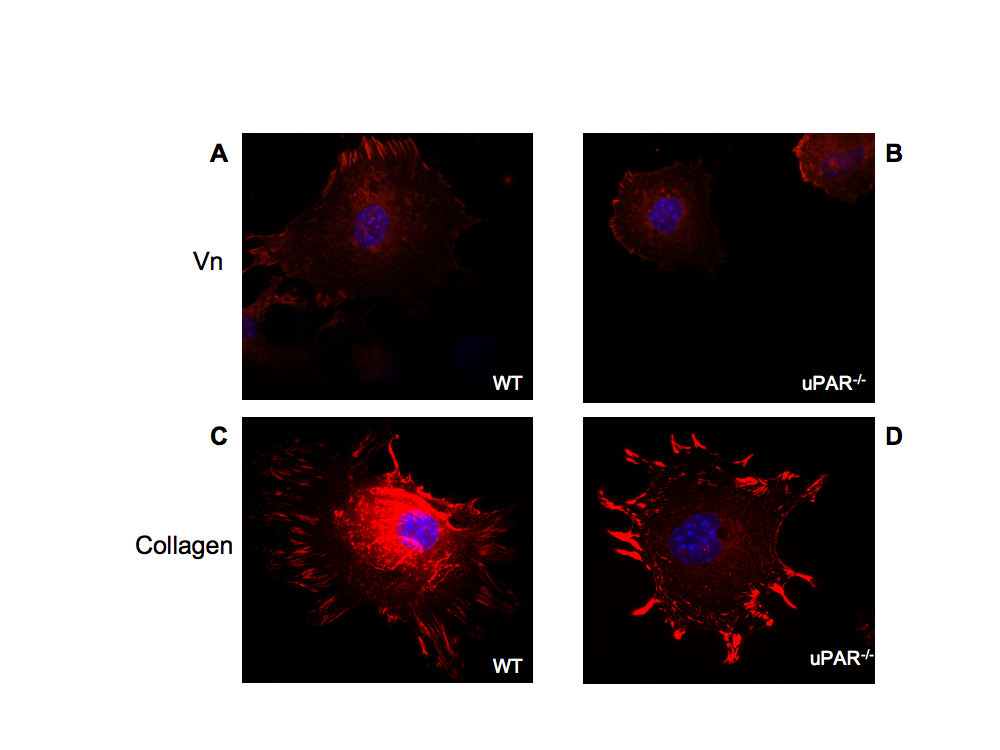

Supplement: Additional file 2 — A Deficiency of uPAR Alters Endothelial Angiogenic Function and Cell Morphology. Figure S2. Perinuclear localization of vinculin in WT and uPAR-/- ECs when plated on Vn: WT and uPAR-/- cells adherent on Vn-coated and collagen-coated oncyte wells for 4 hr were stained with vinculin (red), and DAPI nuclear stain (blue) and images acquired using a 100× objective. It was observed that when WT (A) and uPAR-/- (B) ECs are plated on Vn, vinculin is localized around the nucleus. When WT (C) and uPAR-/- (D) ECs were plated on collagen, vinculin was localized on focal adhesion points. [file 2045-824X-3-10-S2.TIFF]

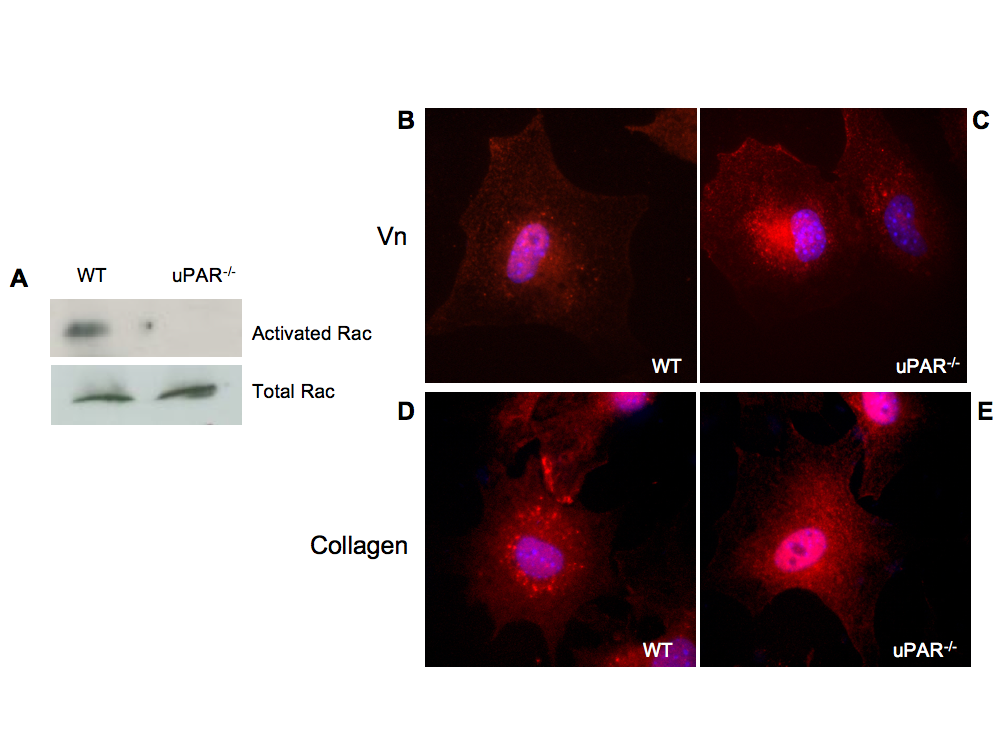

Supplement: Additional file 3 — A Deficiency of uPAR Alters Endothelial Angiogenic Function and Cell Morphology. Figure S3. Effect of uPAR deficiency on Rac activity and RhoA localization: (A) Cell lysates (800 μg) from WT and uPAR-/- ECs adherent on Vn for 4 hr were assayed to determine Rac activity. PAK-1 PBD agarose bound to Rac-GTP was fractionated on a 12% SDS-PAGE gel followed by immunoblotting for Rac. It was observed that uPAR-/- ECs lack endogenous Rac activity compared to WT cells even though total Rac levels were similar. (B, C, D, E) WT and uPAR-/- cells adherent on collagen- or Vn-coated oncyte wells for 4 hr were stained with an antibody to RhoA (red), and DAPI nuclear stain (blue) and images acquired using a 100× objective. It was observed that in WT cells on Vn (B) RhoA is concentrated in a 'halo' around the nucleus while in uPAR-/- ECs (C) RhoA is localized along the membrane. (D) WT cells on collagen demonstrated cytosolic punctate staining of RhoA. (E) Perinuclear localization of RhoA is observed in uPAR-/- cells adherent on collagen. [file 2045-824X-3-10-S3.TIFF]
